# HIV-1 Env associates with HLA-C free-chains at the cell membrane modulating viral infectivity

**DOI:** 10.1038/srep40037

**Published:** 2017-01-04

**Authors:** Michela Serena, Francesca Parolini, Priscilla Biswas, Francesca Sironi, Almudena Blanco Miranda, Elisa Zoratti, Maria Teresa Scupoli, Serena Ziglio, Agustin Valenzuela-Fernandez, Davide Gibellini, Maria Grazia Romanelli, Antonio Siccardi, Mauro Malnati, Alberto Beretta, Donato Zipeto

**Affiliations:** 1 Department of Neurosciences, Biomedicine and Movement Sciences, University of Verona, Strada le Grazie 8, 37134, Verona, Italy; 2 IRCCS Ospedale San Raffaele, Via Olgettina 60, 20132, Milan, Italy; 3 University Laboratory of Medical Research, Piazzale L. A. Scuro 10, 37134 Verona, Italy; 4 Laboratorio de Inmunología Celular y Viral, Unidad de Virología IUETSPC, Unidad de Farmacología, Sección de Medicina, Facultad de Ciencias de la Salud, Universidad de La Laguna (ULL), Campus de Ofra s/n, 38071, Tenerife, Spain; 5 Department of Diagnostics and Public Health, University of Verona, Strada le Grazie 8, 37134, Verona, Italy.

## Abstract

HLA-C has been demonstrated to associate with HIV-1 envelope glycoprotein (Env). Virions lacking HLA-C have reduced infectivity and increased susceptibility to neutralizing antibodies. Like all others MHC-I molecules, HLA-C requires β_2_-microglobulin (β_2_m) for appropriate folding and expression on the cell membrane but this association is weaker, thus generating HLA-C free-chains on the cell surface. In this study, we deepen the understanding of HLA-C and Env association by showing that HIV-1 specifically increases the amount of HLA-C free chains, not bound to β_2_m, on the membrane of infected cells. The association between Env and HLA-C takes place at the cell membrane requiring β_2_m to occur. We report that the enhanced infectivity conferred to HIV-1 by HLA-C specifically involves HLA-C free chain molecules that have been correctly assembled with β_2_m. HIV-1 Env-pseudotyped viruses produced in the absence of β_2_m are less infectious than those produced in the presence of β_2_m. We hypothesize that the conformation and surface expression of HLA-C molecules could be a discriminant for the association with Env. Binding stability to β_2_m may confer to HLA-C the ability to preferentially act either as a conventional immune-competent molecule or as an accessory molecule involved in HIV-1 infectivity.

During the HIV-1 budding process from the cell membrane, Major Histocompatibility Complex (MHC) class I and II molecules are incorporated into the virions together with other cell proteins. A higher number of MHC molecules than envelope (Env) trimers has been reported to be present in HIV-1 virions^1^. Incorporation of cell membrane proteins into HIV-1 envelope is not dependent on their relative amount at the cell membrane since some highly expressed proteins such as CD4, CD45, CCR3, CCR5 or CXCR4 are not incorporated^2^. It has been reported that MHC-I negative cell lines are not competent for the replication of primary HIV-1 isolates^3^ and that HLA-C expression in these cells rescues their HIV-1 replication competence. In addition, it was demonstrated that HLA-C induces changes in the viral envelope protein conformation, including an enhanced presentation of epitopes normally exposed upon CD4 binding^3^ and that HLA-C incorporation into HIV-1 virions reduces their susceptibility to neutralizing antibodies^3^. The specific association between HLA-C and Env has been confirmed in fusion complexes, where the recruitment of HLA-C molecules has been reported within CD4-CCR5-gp120/gp41 complexes, formed on cells during the process of HIV-1-induced cell-to-cell fusion^4^. The same study demonstrated that fusion efficiency is reduced in HLA-C negative cells and that pseudoviruses produced in HLA-C silenced cells are significantly less infectious than those produced in HLA-C expressing cells^4^. Another study demonstrated that HIV-1 infection of peripheral blood lymphocytes requires HLA-C expression, offering an explanation to the specific down-regulation of HLA-A and HLA-B, but not HLA-C, by HIV-1 Nef 5. In 2007 a genome wide association study (GWAS) of the major genetic determinants for HIV-1 host control identified a polymorphism 35 Kb away from the HLA-C transcription initiation (−35 SNP, rs9264942), which has been associated with differences in HLA-C expression levels^6^. Subsequently, it has been reported that the −35 SNP is not the causal variant responsible for the differential HLA-C expression, but rather it is in linkage disequilibrium with another polymorphism at position 263 downstream the HLA-C stop codon (rs67384697)^7^. This polymorphism regulates the binding of the miRNA148a to the target site. As a consequence, HLA-C surface expression appears lower for those alleles which bind miRNA148a, and higher for those alleles escaping this specific post-transcriptional regulation^7^. Consistent with these findings, low expression alleles such as C * 04 and C * 07 have been associated with a more rapid progression toward AIDS than high expression alleles, such as C * 02, C * 06, and C * 12^8^. Consequently, low expression and high expression alleles are also defined as non protective and protective alleles, respectively. Cytotoxic T lymphocytes (CTLs) depletion studies in rhesus macaques clearly demonstrated that CTLs play a critical role in control of HIV-1 infection^9^. It has been proposed that higher HLA-C expression levels could lead to a better antigen presentation to CTLs, explaining the slower progression toward AIDS. In a recent work it has been demonstrated that, in most primary HIV-1 clones, Vpu is able to down-regulate HLA-C but not HLA-A and HLA-B, thus escaping the HLA-C restricted CTLs response, possibly depending on the prevailing host immune pressure: natural killer (NK) versus CTL^10^. Adding complexity to this matter, a recent study failed to confirm the association between HLA-C cell surface expression and the −35 Kb SNP; rather, a high-allelic variability in HLA-C mRNA expression has been demonstrated, suggesting that the control of HLA-C expression might be more complex than expected^11^. MHC-I proteins are heterotrimers composed of a membrane-bound heavy chain, non-covalently linked to an invariant light chain, called β_2_-microglobulin (β_2_m), plus a short cytoplasmic peptide, about 8-11 amino acids long, mostly derived from the degradation of intracellular proteins. MHC-I molecules present these peptides to CD8^+^ T-cells, which survey the body for the presence of foreign material, killing those cells displaying pathogen-derived antigens on their MHC-I molecules^12^. Nevertheless, the amount of HLA-C at the cell surface is about 10-fold lower than that of HLA-A or HLA-B^13^, questioning its effective role in CTL-mediated responses.

HLA-C is thus involved in HIV-1 infection through different and apparently opposite mechanisms: a) the increase of CTLs recognition leading to the lysis of HIV-1 infected cells, b) the inhibition of NK cell recognition, leading to the enhancement of virion infectivity and c) the association with the HIV-1 envelope protein^14^.

In the present work we investigated the association between HIV-1 Env and HLA-C, demonstrating that Env and HLA-C free heavy chains associate at the cell surface. In addition, using the Clustered Regularly Interspaced Short Palindromic Repeats/CRISPR-associated Protein 9 (CRISPR/Cas9) technique to develop β_2_m negative cell lines, we observed that the infectivity of HIV-1 Env-pseudotyped viruses is reduced in the absence of β_2_m, suggesting that HLA-C molecules involved with Env interaction need to be properly folded around β_2_m, which then allows their translocation at the cell membrane.

## Results

### Surface expression of L31 reactive HLA-C molecules is increased in HIV-1 chronically infected cell lines

**Figure 1:**
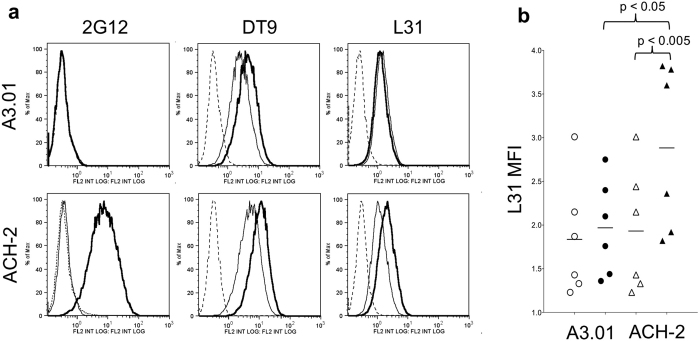
Cytofluorimetric analysis of A3.01 and ACH-2 cell lines. (**a**) Dashed line: negative control (secondary antibody fluorescence); thin line: unstimulated cells; thick line: cells stimulated with TNF-α for 24 h. 2G12, DT9 and L31 antibodies detect HIV-1 Env, HLA-C heterotrimers and HLA-C free chains at the cell membrane, respectively. Following TNF-α stimulation, ACH-2 cells express HIV-1 Env glycoprotein (2G12 antibody); HLA-C heterotrimers are upregulated in both cell types (DT9 antibody); HLA-C free chains are upregulated only in ACH-2 cells (L31 antibody). (**b**) **○**: unstimulated A3.01 cells; **●**: TNF-α stimulated A3.01 cells; **△**: unstimulated ACH-2 cells; **▲**: TNF-α stimulated ACH-2 cells. Data from 6 independent experiments are shown as mean fluorescence intensity (MFI) of L31 staining. L31 fluorescence results to be significantly different between TNF-α stimulated ACH-2 cells (chronically infected by HIV-1) and A3.01 cells (parental cell line, uninfected) (p = 0.01), and between TNF-α stimulated and unstimulated ACH-2 cells (p = 0.002). p values were calculated by two-way ANOVA comparing ACH-2-TNF-α with A301-TNF-α and ACH-2-TNF-α with ACH-2-unstimulated.

**Figure 2:**
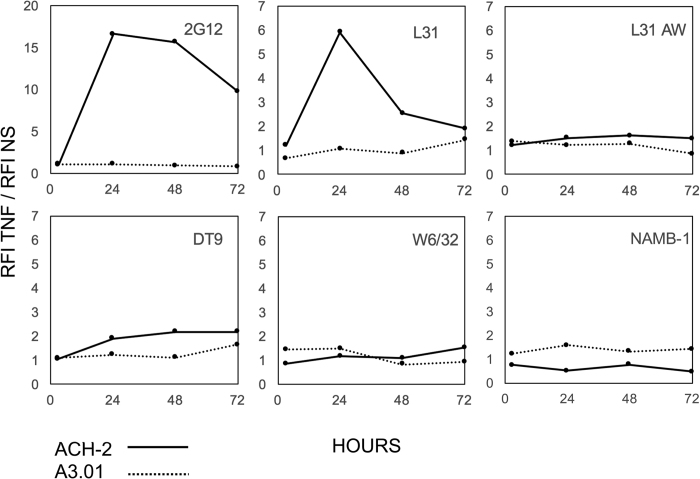
Time course expression analysis of HIV-1 Env, MHC-I and β_2_m in TNF-α stimulated and unstimulated A3.01 and ACH-2 cells. Cells were stained after 3, 24, 48 and 72 h of culture. Solid line: ACH-2 HIV-1 infected cells; dotted line: parental A3.01 cells. RFI (Relative Fluorescence Intensity) = (Mean Flourescence Intensity (MFI) sample − MFI control)/MFI control. Data are shown as fluorescence fold change between RFI of TNF-α stimulated and unstimulated cells. The presence of HLA-C free chains at the cell surface appears to be dependent on the presence of HIV-1 Env; expression of both Env and HLA-C free chains decreases at later times (48 and 72 h post TNF-α stimulation, panel 2G12 for Env and panel L31 for HLA-C free chains). No changes in the expression of total HLA-C (L31 AW panel, after an acid wash to remove β_2_m), HLA-C heterotrimers (DT9 panel), MHC-I (W6/32 panel) or β_2_m (NAMB-1 panel) are observed.

**Figure 3:**
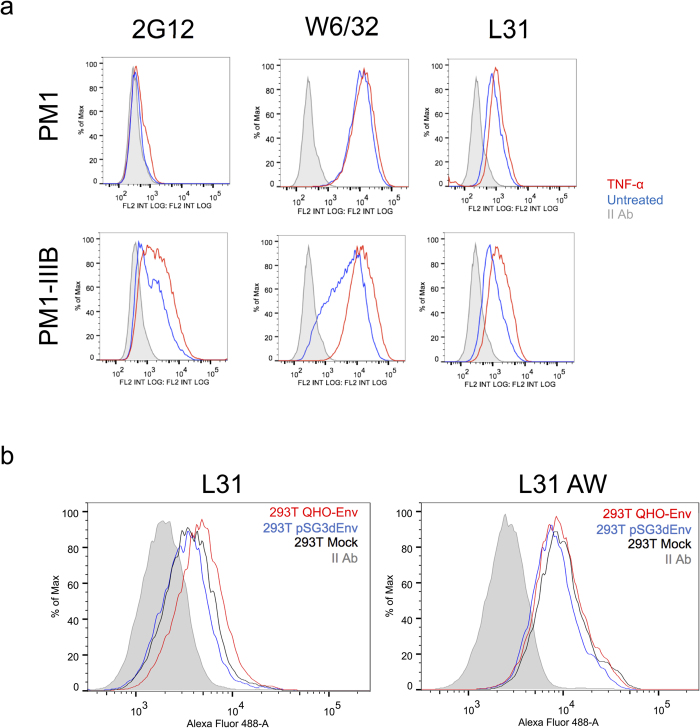
HIV-1 Env expression correlates with HLA-C free chains upregulation. (**a**) Black line: negative control (secondary antibody fluorescence); blue line: PM1 unstimulated cells; red line: PM1 cells stimulated with TNF-α for 48 h. In the absence of stimulation, HIV-1 (IIIB strain) infection (2G12 staining) induces the downregulation of MHC-I molecules (W6/32 staining) and a slight increase of HLA-C free chains molecules at the cell surface (L31 staining), compared to the uninfected cells. After TNF-α stimulation, the HIV-1 replication is increased and the expression of MHC-I molecules at the cell membrane is restored. The increased HIV-1 Env expression correlates with a higher presence of HLA-C free chains molecules at the cell surface of the infected cells, compared to the uninfected ones. (**b**) Shaded curve: negative control (secondary antibody fluorescence); black line: 293T transfected with pcDNA3 (Mock); blue line: 293T transfected with pSG3^Δenv^; red line: 293T transfected with HIV-1 QHO-Env plasmid. L31 staining indicates an increase of HLA-C free chains at the cell membrane of cells transfected with HIV-1 Env, compared to the the transfection with pcDNA3 or pSG3^Δenv^ plasmids. On the contrary, after acid wash (L31 AW) no differences in the amount of total HLA-C molecules were detected.

ACH-2 is a T-lymphocytic cell line chronically infected by HIV-1, derived from the A3.01 uninfected parental cell line^15^. HIV-1 expression can be activated in ACH-2 cells by TNF-α stimulation, through the induction of transcription factors binding the NF-kB site in the HIV-1 LTR region^16^. Both A3.01 and ACH-2 cells were treated with TNF-α and assessed for HIV-1 expression by flow cytometry using the anti-gp120 2G12 monoclonal antibody. A clear fluorescence increase was evident in ACH-2 cells, revealing a marked HIV-1 reactivation (Fig. 1a). Cells were stained in parallel with the DT9 antibody that recognizes HLA-C heterotrimers (HLA-C heavy chain, β_2_m and peptide)^17^ and with the L31 antibody, recognizing β_2_m-free HLA-C molecules^18^. Following TNF-α stimulation, cytofluorimetric analysis showed that β_2_m-associated HLA-C molecules (DT9 reactive) were upregulated in both A3.01 and ACH-2 cells. Conversely, upregulation of HLA-C free chains (L31 reactive) was observed only in HIV-1-infected ACH-2 cells. As shown in Fig. 1b, L31 fluorescence in ACH-2 cells was significantly different between TNF-α stimulated and unstimulated cells (p = 0.002), as well as towards the stimulated parental A3.01 cells (p = 0.01). Upregulation of DT9-reactive HLA-C molecules after TNF-α stimulation in both cell types is expected, since TNF-α as well as interferons are known modulators of expression of MHC-I molecules in many cell types^19^. Conversely the selective cell membrane increase of HLA-C free chains by HIV-1 reactivation is an intriguing original finding. A time course analysis of HIV-1 Env, MHC-I and β_2_m expression was conduced in A3.01 and ACH-2 cells, comparing the RFI between TNF-α stimulated and unstimulated cells (Fig. 2). The results showed that the expression of HLA-C free chains (L31 antibody) at the cell surface correlated with the expression of HIV-1 Env (2G12 antibody), both decreasing at later time points. No changes were instead detected in the expression of total HLA-C molecules (L31 antibody after acid wash), HLA-C heterotrimers (DT9 antibody), MHC-I (W6/32 antibody) or β_2_m (NAMB-1 antibody) molecules. The correlation between HIV-1 infection and HLA-C free chains expression was also observed in an infection model using a different human T-lymphocytic cell line, PM1, chronically infected with HIV-1 III-B (Fig. 3a). PM1-IIIB cells have a constitutive baseline production of virus higher than ACH-2, as visualized by 2G12 staining of unstimulated cells and their downmodulation of MHC-I molecules (W6/32 antibody). When PM1-IIIB cells were stimulated with TNF-α, an increase of HLA-C free chains at the cell surface (L31 antibody) was observed (as in ACH-2 cells), which was barely detected in uninfected PM1 cells, further confirming a role of HIV-1 in promoting the formation of HLA-C free chains at the cell membrane. The mean RFI of L31 staining of three independent experiments was 3.2 and 6.5 in TNF-α-PM1 and TNF-α-PM1-IIIB cells, respectively.

Of interest, TNF-α stimulation counteracted the downregulation of MHC-I molecules induced by HIV-1 infection in PM1-IIIB cells. Given the correlation between increased HLA-C free chains and HIV-1 virion production in both ACH-2 and PM1-IIIB cellular models, we sought to investigate which HIV-1 component was involved in this phenomenon, starting from Env. We thus transfected 293T cells with an HIV-1 Env plasmid and a plasmid encoding an Env defective full length HIV-1 genome (pSG3^Δenv^) (Fig. 3b). An increase of L31 staining was detected after transfection of HIV-Env and not after transfection of the pSG3^Δenv^ plasmid, whereas no differences were detected in L31 staining after acid wash. These data suggest that the observed increase of HLA-C free chains at the cell membrane is not due to an increased HLA-C expression level or membrane translocation, but rather it may reveal a switch in HLA-C conformation from heterotrimer to free chain on the cell membrane in the presence of the HIV-1 Env protein.

### None of the HIV-1 proteins is responsible for the appearance of HLA-C free chains at the cell surface

**Figure 4:**
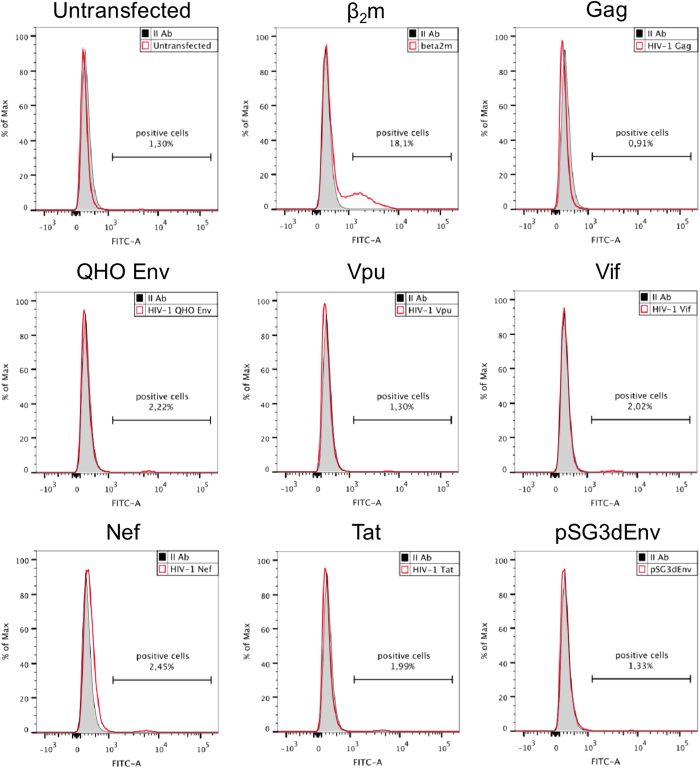
Cytofluorimetric analysis of β_2_m negative 293T cells, after transfection with different HIV-1 genes. Shaded curve: negative control (secondary antibody fluorescence); red line: L31 labelled cells. After transfection with plasmids encoding different HIV-1 proteins, cells were labelled with the L31 antibody, after acid wash. As a positive control cells were transfected with β_2_m. Cytofluorimetric analyses indicate that only β_2_m transfection is able to restore HLA-C at the cell membrane of β_2_m negative cells. Neither the presence of Env, nor the presence of other HIV-1 proteins is sufficient to translocate HLA-C to the cell surface.

**Figure 5:**
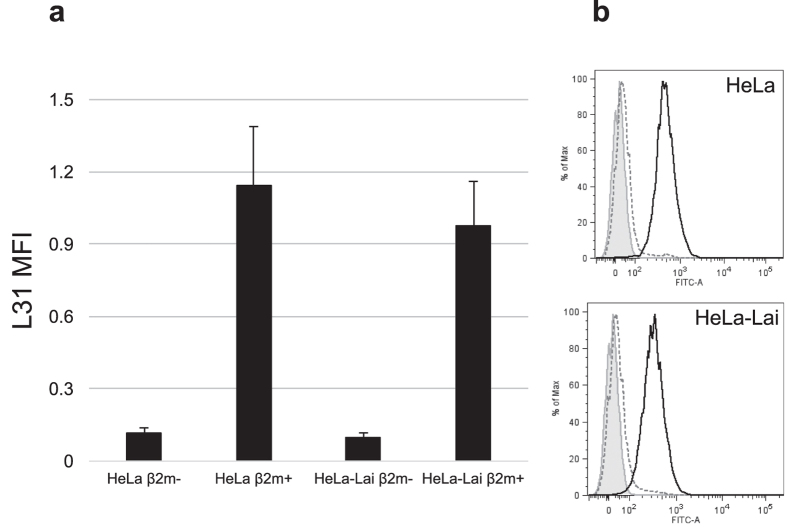
The presence of HLA-C free-chains at the cell membrane is dependent on β2m expression. (**a**) L31 labelling, after acid wash, indicates that both HeLa and HeLa-Lai β_2_m negative cells (β_2_m−) are unable to express HLA-C molecules at the plasma membrane while the parental cell lines (β_2_m+) normally translocate HLA-C to the cell surface. The error bars represent standard errors from the mean calculated from quadruplicate determinations. (**b**) Shaded curve: negative control (secondary antibody fluorescence); dashed line: β_2_m− cells; solid line: β_2_m+ cells. HLA-C membrane expression is detected only in the parental β_2_m positive cells.

To investigate if any of the HIV-1 proteins is instead responsible for the translocation of HLA-C free chains at the cell membrane, plasmids encoding Gag, Env, Vpu, Vif, Nef, Tat, as well as pSG3^Δenv^ (containing the Env defective full length HIV-1 genome) were transfected in CRISPR/Cas9 β_2_m knockout 293T cells. Cells were treated with an acid wash, before labelling with L31 antibody, in order to exclude that the potential intracellular association between HLA-C and any of the tested HIV-1 protein could mask the L31 epitope^18^. Control transfection with β_2_m expressing vector increased the surface expression of HLA-C free chains, while no HLA-C upregulation was detected on the membrane of cells transfected with all the HIV-1 proteins encoding constructs (Fig. 4). Moreover, we prepared β_2_m knockout variants of both HeLa and HeLa-Lai cell lines using the CRISPR/Cas9 technique. Both parental and β_2_m negative cells were analysed by flow cytometry for the presence of HLA-C free chains at the cell surface using the L31 antibody (Fig. 5). We observed that both HeLa and HeLa-Lai β_2_m negative cells lacked the expression of HLA-C at the cell membrane, indicating that the presence of HIV-1 Env in HeLa-Lai was not sufficient for HLA-C translocation, which strictly requires β_2_m for MHC-I assembly and transportation to the surface, where the presence of Env might favour the formation of HLA-C free chains.

### HIV-1 Env and HLA-C interaction: BiFC assay

**Figure 6:**
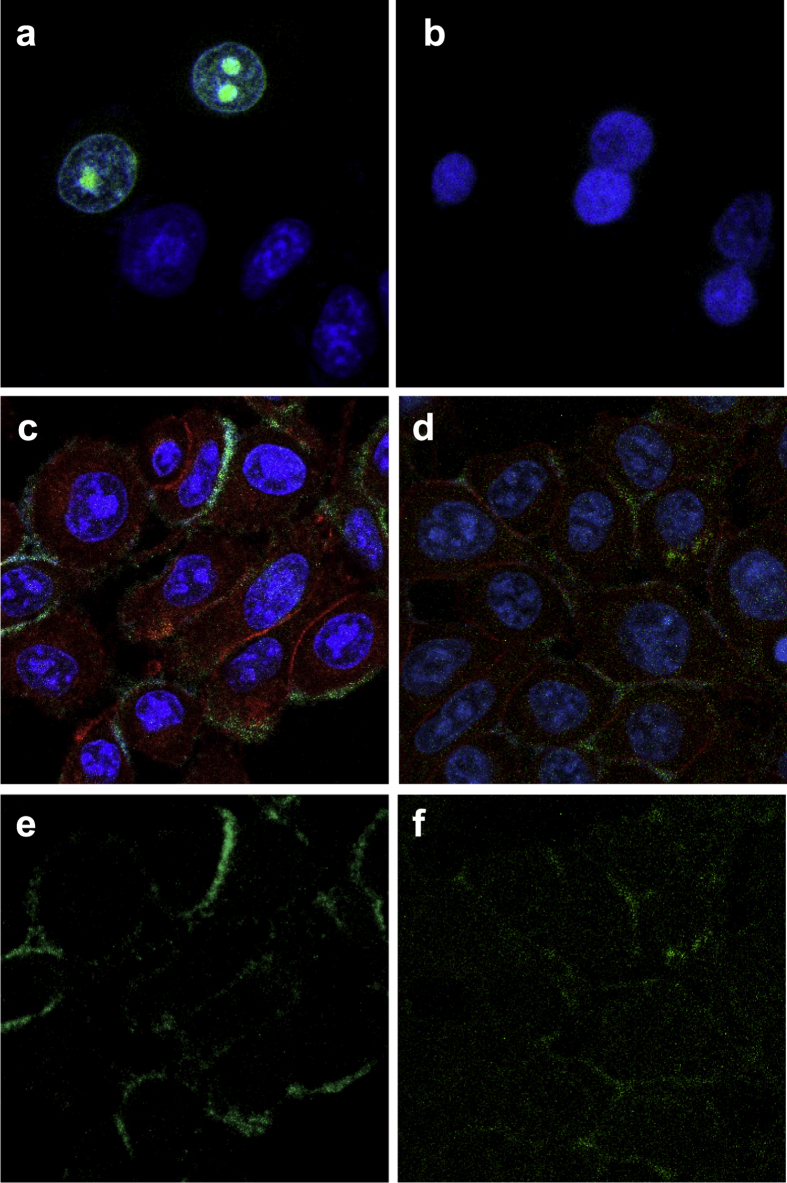
BiFC assay between Env and HLA-C in HeLa cells. (**a**) Nuclear complementation between Jun-Yn and Fos-Yc was assessed as a positive control (green signal). (**b**) Complementation between Env-Yn and Fos-Yc was assessed as a negative control. (**c**,**e**) Transfection of Env-Yn and Env-Yc leads to the BiFC signal (green), indicating Env oligomerization at the cell membrane. (**d**,**f**) Transfection of Env-Yn and HLA-C-Yc leads to the BiFC signal (green) indicating the Env/HLA-C interaction at the cell membrane. In panels c and d β_2_m is labelled in red.

Previous studies demonstrated the association between Env and HLA-C^3^,^4^. To analyse Env and HLA-C interaction, the Bimolecular Fluorescence Complementation (BiFC) assay was exploited. BiFC assay is based on the association between two non-fluorescent fragments of a fluorescent protein which have low affinity for each other, but when they are fused to interacting proteins, the fluorophore is reconstituted and a fluorescent signal is produced by bimolecular complementation^20^. Several constructs were developed, cloning the N- and C- terminal fragments (Yn and Yc) of the Yellow Fluorescent Protein (YFP) to the C-terminus of Env and HLA-C proteins. The system was validated in HeLa cells, assessing the complementation between Jun-Yn and Fos-Yc, which resulted in a green signal in the nucleus (Fig. 6a), as previously described^21^. Since it is known that Fos is efficiently translocated from cytoplasm to the nucleus only after interacting with Jun through their leucine zippers and by the use of the nuclear localization signal of Jun^22^, the nuclear BiFC signal confirms the Fos/Jun association. For the same reason when Fos-Yc, which in the absence of Jun localizes in the cytoplasm, was co-transfected with Env-Yn and the result was a BiFC negative signal (Fig. 6b). Finally, HeLa cells were co-transfected with Env-Yn and Env-Yc and stained with the anti-β_2_m antibody (red signal). The BiFC green signal was detected at the plasma membrane, due to Env-Env interaction (Fig. 6c,e)^23^. Similarly, co-transfection of Env-Yn and HLA-C-Yc reconstituted the YFP fluorophore at the cell membrane (Fig. 6d,f). All the z-stacks from a single cell were further investigated (see Supplementary Movie S1). The BiFC green signal was not widespread, but rather polarized in specific membrane clusters. The Env/HLA-C complex did not appear to co-localize with β_2_m, indicating that HLA-C molecules may associate at the cell membrane either with Env or with β_2_m, resulting in green or red fluorescence, respectively. The absence of a clear co-localization signal suggests that the association between Env and HLA-C involves HLA-C free chains.

### HIV-1 Env interacts with HLA-C at the cell membrane

**Figure 7:**
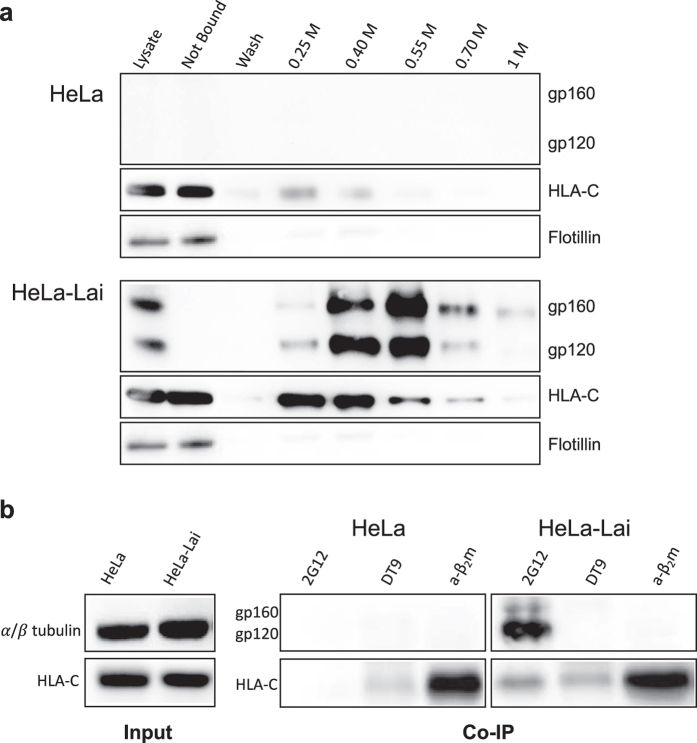
Env and HLA-C are associated at the cell surface. (**a**) HeLa and HeLa-Lai cells were treated with the cell membrane insoluble DTSSP cross-linker. Proteins were purified on GN lectin columns by elution with increasing concentrations (from 0.25 to 1 M) of methyl α-D-mannopyranoside. Purified complexes heavier than 100 kDa were analysed by western blot. Env purification is evident in HeLa-Lai cells and not in control HeLa cells. HLA-C molecules, containing few mannoses, are eluted in the first fractions from HeLa cells, while they are co-purified in higher amounts in the presence of Env (HeLa-Lai). No differences in flotillin-1 purification, used as negative control, were detected. (**b**) Membrane protein complexes from HeLa and HeLa-Lai cells were immunoprecipitated with 2G12 (anti-Env), DT9 (anti-HLA-C) and a-β_2_m (anti-β_2_m) antibodies. Western blot analysis of immunoprecipitated samples shows that HLA-C is associated either with β_2_m or with Env. HLA-C and α/β-tubulin expression were detected as loading control.

To further investigate the association between Env and HLA-C at the cell membrane, HeLa-Lai cells were treated with the cell membrane insoluble, thiol-cleavable DTSSP reagent^24^, which was used to crosslink protein complexes at the cell surface. Proteins were purified on a GN lectin column^25^ which specifically binds D-mannose groups present on the HIV-1 Env glycoprotein and several fractions were obtained by elution with increasing concentrations of methyl α-D-mannopyranoside. Purified complexes heavier than 100 kDa were analysed by western blot after reduction (Fig. 7a). As a negative control, identical amounts of proteins from HeLa cells were analysed following the same procedure. To exclude any excessive cross-linking of membrane proteins due to DTSSP treatment, flotillin-1, a membrane protein, was tested: no specific copurification in the presence of HIV-1 Env was observed, and no differences in its purification were detected between the two cell lines. HLA-C molecules produced in HeLa cells were eluted in the first fractions as they contain few mannoses^26^, whereas HLA-C molecules produced in HeLa-Lai cells were co-purified, in parallel with Env, in higher amounts and in all the fractions. In addition, co-immunoprecipitation of protein complexes from the cell surface of both Hela and HeLa-Lai cells with the 2G12 antibody (anti-Env), further confirmed the association between Env and HLA-C (Fig. 7b), in agreement with previously reported data^3^. Incubation with the DT9 antibody did not result in Env immunoprecipitation, excluding the involvement of HLA-C heterotrimers in the association with Env. Overall, these results indicated that HLA-C free chains are co-purified with Env at the cell membrane.

### Functional effect of β_2_m on HIV-1 infectivity

**Figure 8:**
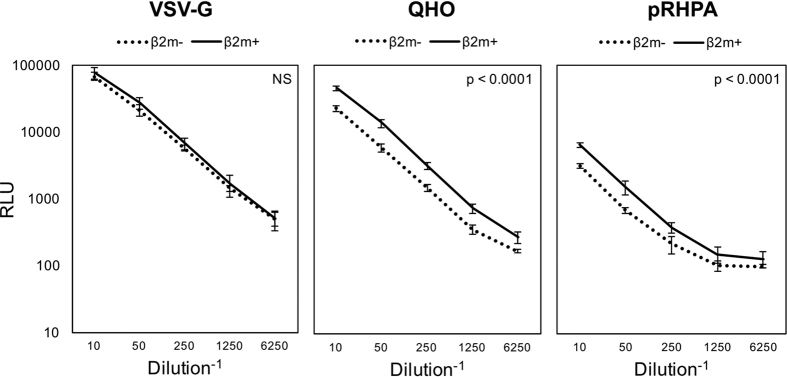
Luciferase-based HIV-1 pseudoviruses infectivity assay on TZM-bl cells. Pseudoviruses produced in parental 293 T β_2_m+ cells (solid line) are significantly more infectious than those produced in 293 T β_2_m− cells (dotted line). This is observed for two HIV-1 Env pseudotyped viruses (QHO and pRHPA) (p < 0.0001) and not for an unrelated pseudotyped virus (VSV-G). Data are expressed as Relative Luminescence Units (RLU). The error bars represent standard deviations from the mean calculated from quadruplicate determinations. p values were calculated by two-way ANOVA, comparing β_2_m+ and β_2_m− pseudoviruses.

Previous results showed that HIV-1 envelope glycoprotein specifically associates with HLA-C, improving the ability of virions to infect target cells^4^. To discriminate the role of β_2_m in virion infectivity, Env-pseudotyped viruses (QHO and pRHPA) were produced in parental 293T and in CRISPR/Cas9 β_2_m negative 293T cells. An infectivity assay was performed with TZM-bl cells, in which the luciferase expression signal reflects virus infectivity^27^. Equivalent amounts of pseudoviruses, determined by p24 titration, were used. Luciferase expression resulted significantly higher following infection with pseudoviruses originating from 293T-β_2_m positive cells than with the ones derived from 293T-β_2_m negative cells (p < 0.0001) (Fig. 8). Analysis of the interpolating curve, best fitting the Relative Luminescence Units (RLU) values, indicated that in the absence of β_2_m, the virus was about 3 times less infectious. No difference in infectivity was observed using Vesicular Stomatitis Virus (VSV) G protein pseudotyped viruses produced in both 293T cells types. These results suggested that β_2_m increases HIV-1 infectivity ensuring HLA-C translocation at the cell surface, where the association with Env occurs.

## Discussion

HLA-C molecules are selectively incorporated into the HIV-1 envelope during virus budding from the host cell membrane^2^. Several evidences of the interaction between Env and HLA-C indicate that the presence of HLA-C is necessary to facilitate HIV-1 infection. The two molecules have been found associated and in the absence of HLA-C both fusion efficiency and virus infectivity are reduced^3^,^4^,^5^. In this work, a significant up-regulation of surface-HLA-C free chains, reactive to the L31 antibody^18^ was observed in HIV-1 chronically infected cells (ACH-2) following virus reactivation by TNF-α stimulation. This was not observed in the corresponding uninfected parental cell line (A3.01). On the contrary, cellular staining with the DT9 antibody, which detects HLA-C heterotrimers^17^, did not reveal any difference between the two cell lines, suggesting that HIV-1 reactivation selectively enhances HLA-C free chains expression at the cell surface. Moreover, the amount of HLA-C free chains at the cell surface correlated with the expression of HIV-1 Env, decreasing after 48 and 72 h from TNF-α stimulation. On the contrary, no differences in the cell membrane expression of total HLA-C molecules, HLA-C heterotrimers, MHC-I or β_2_m were detected, indicating that HIV-1 infection may preferentially increase the amounts of HLA-C free-chain conformation. This observation was further confirmed in another cell line (PM1), where the TNF-α-induced increase of HIV-1 replication correlated with the upregulation of HLA-C free chains at the cell membrane. In addition we observed that Env-transfected 293T cells expressed more HLA-C free chains on their membrane than 293T cells transfected with the Env defective full length HIV-1 genome (pSG3^Δenv^ plasmid), suggesting that Env may promote the formation of HLA-C free chains at the cell surface. HIV-1 infection does not directly cause upregulation of HLA-C, neither at the expression level nor by modulating its stability or membrane translocation. We hypothesize that the presence of HIV-1 Env favours the dissociation of β_2_m from HLA-C, thus resulting in a higher amount of HLA-C free chain molecules at the plasma membrane, but this point requires further investigation. β_2_m is essential to translocate HLA-C at the cell surface, where β_2_m light chain might dissociate from HLA-C heavy chain and the association of HLA-C free chain with Env may take place. β_2_m is necessary for ensuring HLA-C translocation at the cell surface, indicating that HLA-C molecules strictly require β_2_m for reaching the plasma membrane, following the classical MHC-I pathway and that neither the presence of Env, nor the presence of other HIV-1 proteins, is responsible for HLA-C free-chains translocation. Our results with the BiFC technique provided experimental evidence of a very close association between Env and HLA-C free chains at the cell membrane. The association was further confirmed both by proteins purification on GN lectin columns after membrane proteins crosslinking and by co-immunoprecipitation of membrane protein complexes from Hela and HeLa-Lai cells. HLA-C molecules are characterized by a post-translational biosynthetic bottleneck due to an inefficient assembly with the peptide, which leads to accumulation in the endoplasmic reticulum of both β_2_m-free and β_2_m-associated folding intermediates^28^. This may be the reason why HLA-C could more easily dissociate from β_2_m compared to HLA-A and -B. Due to their long half-life, MHC-I free chains have been shown to cis-associate both with themselves and with a variety of membrane receptors, such as CD3, CD8αβ, CD25, Ly49A and IL-15Rα^2^,^29^,^30^,^31^. It is therefore plausible that HLA-C free chains may also have the propensity to associate with Env. Since β_2_m is essential for the assembly of MHC-I molecules and their translocation to the plasma membrane, we investigated the role of β_2_m in HIV-1 infectivity. In particular, we perfomed an infectivity assay in TZM-bl cells, demonstrating that the infectivity of HIV-1 pseudoviruses produced in β_2_m negative 293T cells was roughly 3 times lower than the infectivity of pseudoviruses produced in the corresponding β_2_m positive parental 293T cells. A similar 3-fold reduction in HIV-1 infectivity was also reported by Cosma *et al*.^3^. Our data are also supported by previous data showing that silencing of HLA-C expression significantly reduces HIV-1 infectivity^4^. This result further confirms the involvement of HLA-C in increasing HIV-1 infectivity^4^, indicating that β_2_m is essential for the translocation of HLA-C to the cell surface where, after β_2_m dissociation, HLA-C free chains might associate with Env. However, since β_2_m knockout in 293T cells also highly affects the expression of other classical and non-classical HLA-Class I molecules, the observed reduction in infectivity of the virions may not be exclusively due to the absence of HLA-C on the cell surface. The experiments described, together with previously reported data^3^,^4^,^5^, demonstrate that HLA-C interacts with Env; in particular in this study we showed that the enhanced infectivity conferred to the virus by HLA-C, previously reported by our group^4^, involves HLA-C free chains. Overall, the data presented in this work support the hypothesis that HLA-C involvement in HIV-1 infection may confer some advantages to the virus. HLA-C may be involved in several HIV-1 processes, such as improving the virion assembly process, reducing the shedding of viral glycoproteins from the cell membrane, stabilizing, through a cis-interaction, the Env proteins on the virion in a favourable conformation for cell attachment and fusion. The presence of HLA-C also reduces HIV-1 virions susceptibility to neutralizing antibodies^3^. On the other hand, it has been reported that HLA-C is involved in the immune control of HIV-1 infection, contributing to recognition by CTLs and lysis of HIV-infected cells. Protective genotypes have been correlated with high levels of HLA-C surface expression^6^,^32^: higher HLA-C surface expression has been associated with a stronger control of viral load^33^. A recent study^34^ reported that the different amounts of surface-expressed HLA-C is dependent on the allelic variant and confirmed the correlation between high HLA-C levels and control of HIV-1 viremia. However, HLA-C does not efficiently present antigen to CTLs, while it is an extremely good ligand for KIR receptors, protecting target cells from lysis mediated by NK cells^35^. This may imply that other properties and functions of HLA-C alleles could be involved^14^. Recently, the relation between HLA-C alleles and expression levels has been questioned by a study in which the reported association between HLA-C cell surface expression and the −35 Kb polymorphism was not confirmed. Rather a high-allelic variability in HLA-C mRNA expression was reported^11^. The protective role of highly expressed alleles towards infection is in apparent contrast with our results, which show a role for the Env/HLA-C association in enhancing viral infectivity. Interestingly, in this work we showed that the interaction between HIV-1 and HLA-C selectively involves HLA-C free chains (L31 reactive). HLA-C expression observed by Apps *et al*.^34^ was assessed using a different antibody (DT9), which recognizes β_2_m-associated HLA-C and cross-reacts with HLA-E^36^. In our study, the L31 antibody, specific for the HLA-C free chains, allowed the definition of the HLA-C conformation as an important discriminatory factor for the association with HIV-1 Env. This may confer to HLA-C allelic variants a selective capacity to function as accessory molecules in the process of virus assembly and budding^14^. Differences reported among HLA-C alleles might rely on intrinsic differences in their lower or higher ability to bind β_2_m. It is known that murine MHC- I molecules show allelic differences in their interaction with β_2_m^37^. In addition, it should be taken into consideration the possibility that HIV-1 could have the capacity to directly displace β_2_m from HLA-C and/or bind β_2_m, as previously reported for CMV^38^. β_2_m has been detected on HIV-1 virions^39^, as well as on HTLV-I^40^, echoviruses^41^ and coxsackievirus^42^ viral particles. β_2_m bound to these viruses is involved in modulating viral infectivity as well as in conferring protection from neutralizing antibodies. Furthermore, β_2_m bound to HIV-1 virions is recognized by a specific antibody, R7V, which neutralizes different HIV-1 isolates but does not bind to the cell surface; this epitope appears to be exposed only when β_2_m is bound to HIV-1 particles^43^. In conclusion, the alternative conformations of HLA-C may directly affect the balance between protection and susceptibility outcome. In particular, its prevalence at the cell membrane either as heterotrimer or as free chain could determine whether HLA-C is more advantageous to the host or to the virus, respectively. Despite differences in the expression of HLA-C alleles do exist, due to the action of redundant regulatory mechanisms, a common feature is the poor HLA-C assembly^28^. This conformational instability suggests that HLA-C allotypes weakly binding β_2_m might be mainly present as free chains, resulting in a selective propensity for association with other proteins, including HIV-1 Env. The stability of different HLA-C alleles was assessed in a previous work by measuring the relative abundance of both free and β_2_m-associated HLA-C molecules at the plasma membrane^28^. A correspondence may be observed between highly expressed alleles, according to Apps’ definition^34^, and a more stable association with β_2_m, and between low expressed alleles and prevalence as free chains. In particular, low expressing alleles, such as C * 04 and C * 07, showed a strong prevalence as L31-reactive free chains, while high expressing alleles, such as C * 06 and C * 08, showed an increased β_2_m-association, detected with the W6/32 antibody^28^. A complete characterization of HLA-C conformations on infected cells is required, in relation to their expression level. We are currently attempting to investigate if the different amounts of HLA-C detected at the cell membrane may also be attributed to different predominant conformations. Different HLA-C alleles should also be evaluated for possible differences in the interaction with HIV-1 Env. This study suggests that HLA-C expression may go beyond its immunological role; rather it could have a deeper implication during HIV-1 infection. Our results help to elucidate the interaction between HLA-C and Env and provide new insights into the complex role played by HLA-C in HIV-infection. The understanding of this interaction may assist in the design of new therapeutic strategies aimed at controlling HIV-1 infection.

## Methods

### Cell lines

HeLa and 293T cells were obtained from the American Type Culture Collection (ATCC). HeLa-Lai cells were kindly provided by Dr. Uriel Hazan, Institut Cochin, Paris, France. Briefly, these cells were obtained by transfection of a HIV-1 provirus in which *gag* and *pol* genes were deleted, while *nef* was replaced by the *dhfr* drug resistance^44^. TZM-bl cells were provided by the EU Programme EVA Centre for AIDS Reagents, NIBSC (ARP5011)^45^. A3.01^46^ is a CD4^+^ T-lymphoma cell line, from which the HIV-latently infected cell line (ACH-2) has been derived by *in vitro* infection with HIV-1 IIIB. The viral expression is induced in ACH-2 cells by stimulation with TNF-α^16^,^47^,^48^. PM1 (from Dr. P. Lusso, AIDS Research and Reference Program, Division of AIDS, NIAID, NIH, USA) is a clonal derivative of the human T lymphocytic HUT 78 cell line and PM1-IIIB is the chronically-infected counterpart. All the cell lines were grown at 37 °C in a humidified atmosphere with 5% CO_2._ HeLa, HeLa-Lai, 293T, TZM-bl cell lines were cultured in Dulbecco’s modified Eagle’s medium, high glucose (Euroclone), while A3.01, ACH-2 and PM1 cell lines were cultured in RPMI-1640 medium (Euroclone). Culture medium was supplemented with 10% FBS, 2 mM L-Glutamine, 100 U Penicillin/ml and 100 U Streptomycin/ml (Lonza).

### Vectors

Plasmids coding for HIV-1 Env, R5 strain QHO (ARP2043, Dr. D. Montefiori and Dr. F. Gao)^27^ and R5 transmitted/founder strain pRHPA (ARP2061, Dr. B. H. Hahn and Dr. J. F. Salazar-Gonzalez)^27^ were provided by the EU Programme EVA Centre for AIDS Reagents, NIBSC (Courtesy of NIH AIDS Research and Reference Reagent Programme), as well as the pCV1 plasmid, encoding for HIV-1 Tat (ARP2004, Dr. F. Wong-Staal)^49^. The pSG3^Δenv^ backbone plasmid (catalogue number 11051, Dr. J. C. Kappes and Dr. X. Wu)^50^, the plasmids encoding HIV-1 Vif (pcDNA-HVif, catalogue number 10077, Dr. S. Bour and Dr. K. Strebel)^51^ and the HIV-1 Gag (catalogue number 11468, Dr. M. D. Resh and G. Pavlakis)^52^ were obtained from the NIH AIDS Research and Reference Reagent Program. The plasmid encoding the 91US005 HIV-1 Env was prepared as previously reported^53^. The plasmid expressing the HLA-C (pZeo-HLA-C) was provided by Prof. A. Siccardi (Ospedale San Raffaele, Milan, Italy), while plasmids encoding Nef and Vpu were kindly provided by Dr. M. Pizzato (CIBIO, Trento, Italy)^54^. Plasmids expressing the VSV-G protein (pCMV-VSV-G) and the human β_2_m-microglobulin (pBJ1-human β_2_m) were purchased from Addgene, as well as the pSpCas9(BB)-2A-Puro (PX459) V2.0 vector. The plasmid pCR-Blunt II-TOPO encoding the β_2_m gRNA (crB2M_13) was provided by Dr. C.A. Cowan (Harvard University, MA USA)^55^. Jun-Yn and Fos-Yc plasmids were provided by Prof. Tom K. Kerppola (University of Michigan, Ann Arbor, MI USA).

### Antibodies

The human 2G12 (EVA3064, Dr. D Katinger)^56^ and the mouse CA13 (ARP3119, Ms. C Arnold) antibodies were provided by the EU Programme EVA Centre for AIDS Reagents, NIBSC; the L31^18^, the W6/32^57^ and the NAMB-1 antibodies^58^ were kindly provided by Dr. P. Giacomini (Regina Elena Hospital, Rome, Italy); the DT9 antibody^17^ was a kind gift from A. Fenton-May and P. Borrow (Nuffield Dept. of Clinical Medicine, University of Oxford, UK). The rabbit anti-β_2_m (ab15976) and the rabbit anti α/β-tubulin (# 2148) antibodies were purchased from Abcam and Cell Signaling, respectively. The mouse anti-flotillin-1 antibody (# 610821) was available from BD Transduction Laboratories. As secondary antibodies, the Alexa Fluor 488 conjugated goat anti-human (Southern Biotech), the phycoerythrin-conjugated anti-mouse (Southern Biotech), the Alexa Fluor 488-conjugated goat anti-mouse (Cell Signaling), the Texas red-labelled sheep anti-rabbit (Abcam) and the HRP-conjugated anti-mouse (Promega) were used.

### Transfections

Cells were transfected with the TransIT-LT1 transfection reagent (Mirus Bio), following manufacturer’s instructions. GFP expression was assessed as a transfection efficiency control.

### Development of β_2_m knockout cell lines by CRISPR/Cas9

293T, HeLa and HeLa-Lai cells were co-transfected with the pCR-Blunt II-TOPO β_2_m gRNA^55^ and the pSpCas9 vectors. After puromycin selection, β_2_m negative cells were labelled with the NAMB-1 antibody and sorted using a FACSAria II cell sorter (BD Biosciences, San Josè, CA).

### Flow cytometry analyses

A3.01, ACH-2 and PM1 cells were cultured either in the absence or in the presence of TNF-α (10 ng/ml) for the indicated time points. Cells were stained for 30 minutes at 4 °C with 2.5 μg/ml of DT9, 1 μg/ml of L31 and W6/32, 5 μg/ml of 2G12 and 1:200 dilution of ascitic fluid for NAMB-1. After PBS washes, cells were labelled with phycoerythrin-conjugated anti-mouse or Alexa Fluor 488 conjugated anti-human antibodies. Cells were fixed with 1% formaldehyde prior to acquisition with a Gallios flow cytometer (Beckman Coulter) and analysed using the FlowJo software (TreeStar, San Carlos, CA). Data for the time course analysis between TNF-α stimulated and unstimulated A3.01 and ACH-2 cells were expressed as fluorescence fold change between RFI (Relative Fluorescence Intensity) of TNF-α stimulated and unstimulated cells. β_2_m negative 293 T cells (transfected with the different HIV-1 plasmids), as well as parental and β_2_m negative HeLa and HeLa-Lai cells, were labelled with 1 μg/ml of L31 antibody for 30 minutes at 4 °C, followed by staining with Alexa Fluor 488-conjugated goat anti-mouse antibody. Data were acquired with a FACSCanto flow cytometer (BD Bioscience) and analysed using the FlowJo software (TreeStar, San Carlos, CA). For HLA-C free chains determination (L31 acid wash), cells were treated with RPMI-1640, 20% FBS, pH 2.5 for 3 minutes in ice to displace β_2_m, prior to washes and staining with L31.

### Env-Yc, Env-Yn, HLA-C-Yc, HLA-C-Yn BiFC constructs

The Env-Yn, Env-Yc, HLA-C-Yn, HLA-C-Yc constructs were prepared from *Env* and *HLA-C* sequences from the 91US005 *Env*^53^ and pZeo-HLA-C plasmid, respectively. Gene sequences were amplified by PCR with a forward primer harbouring *Xba*I restriction site (HLA-C: CACC*TCTAGA*ATGCGGGTCATGGCGCCCCG; Env: *TCTAGA*ATGGCAGGAAGAAGCGGAGAC) and a reverse primer, abolishing the protein stop codon, harbouring *Xho*I restriction site (HLA-C: CGA*CTCGAG*TCGGCTTTACAAGCGATGAG, Env: TCC*CTCGAG*CCAGCCCTGTAAATTCTTTGTATT). HLA-C and Env amplification products were cloned in pcDNA 6.2 plasmids (Invitrogen), upstream either the YFP N-terminus (Yn) or the YFP C-terminus (Yc). The Yn and the Yc fragments were obtained by PCR amplification of YFP from pcDNA6.2/N-YFP plasmid (Invitrogen) with primer pairs harbouring *EcoR*I restriction sites (Yn fragment: Fw: TGA*GAATTC*ATGGTGAGCAAGGGCGAG, Rv: ACT*GAATTC*TTACTGCTTGTCGGCCATGATA; Yc fragment: Fw: CAG*GAATTC*AAGAACGGCATCAAGGTG, Rv: ACT*GAATTC*TTACTTGTACAGCTCGTCCATG) to allow cloning in pcDNA6.2. Each construct was verified by sequencing.

### Bimolecular Fluorescence Complementation (BiFC) assay

HeLa cells were transfected with BiFC constructs and then switched to 30 °C for 2 hours to allow the fluorophore maturation^21^. Cells were fixed with 4% paraformaldehyde and labelled with the anti-β_2_m antibody (Abcam). After PBS washing, cells were incubated with Texas red-labelled sheep anti-rabbit antibody and nuclei were stained with Hoechst. Coverslips were mounted with Dako fluorescence mounting medium. The images were captured using a confocal laser-scanning fluorescence microscope Leica TCS SP5 and analysed with the LAS AF software (Leica). For each experiment, the Jun-Yn and Fos-Yc complementation was assessed as a positive control^21^, while the complementation of Fos with either HLA-C or Env was tested as a negative control.

### Env/HLA-C co-purification

HeLa and HeLa-Lai cells were fixed with 1 mM DTSSP (ThermoScientific) according to manufacturer’s instructions. Cells were washed with PBS and resuspended in lysis buffer (2.5 mM Hepes, 145 mM NaCl, 1% Triton, 0.1 mM PMSF). The cell lysate was centrifuged to pellet nuclei and the supernatant was passed over a snowdrop lectin column (Galanthus nivalis lectin, Sigma)^25^,^59^, previously equilibrated with dialysis buffer (2.5 mM HEPES, 145 mM NaCl, pH 7.4, and 0.1% Triton). Proteins were collected in five elution fractions containing increasing concentrations (0.25, 0.40, 0.55, 0.70 and 1 M, one column volume) of methyl α-D-mannopyranoside (Sigma). Each eluted fraction was concentrated using a 100 KDa Amicon Ultra Centrifugal Filter (Millipore). Lysate (25 μg), not bound (25 μg) and 1/4 of the total volume of concentrated fractions were incubated for 10 minutes at 98 °C in the presence of 3% β-Mercaptoethanol, to cleave the DTSSP thiol links, and then analysed by western blot.

### Co-immunoprecipitation of protein complexes at the cell surface

Co-immunoprecipitation was performed as previously described^60^. Briefly, HeLa and HeLa-Lai cells were labelled at the cell surface with 2G12, DT9, or anti-β_2_m (Abcam) antibodies, for 45 minutes at 4 °C. After PBS washing, cells were resuspended in 0.5% NP-40 non-denaturing lysis buffer. Cellular nuclei were removed by centrifugation at 3000 rpm for 3 minutes. Cell surface labelled proteins complexes were incubated at 4 °C with Dynabeads^®^ protein G (Life Technologies) for 45 minutes. Beads were washed and resuspended in elution buffer containing 2% SDS and 40 mM DTT. After boiling at 98 °C, proteins were separated by SDS-PAGE and analysed by western blot.

### Western blot analysis

Immunoblot analysis was performed using the CA13 (1:200) for HIV-1 Env, the L31 (1:200) for HLA-C, the anti-flotillin-1 (1:500) or the anti-α/β-tubulin (1:2000) antibodies, followed by incubation with HRP-conjugated anti-mouse or anti-rabbit antibodies. The signal was developed using the ECL AdvanceTM Western Blotting Detection Kit (Amersham), through the AutoChemi System UVP (BioImaging System).

### Production of Env-pseudotyped viruses and TZM-bl assay

Pseudoviruses expressing the *Rev*/*Env* sequences of different HIV-1 subtype-B strains (QHO and pRHPA) were produced by co-transfecting 293 T cells with the pSG3^Δenv^ backbone plasmid and collecting the pseudoviruses-containing supernatant after 48 hours, as previously described^27^. VSV-G was used as a control. Pseudoviruses were quantified using the p24 ELISA kit (Aalto Bio Reagents Ltd, Dublin, Ireland) and the same amount of virus was used to infect 1 × 10^4^ TZM-bl cells in the presence of 20 μg/ml DEAE-dextran^27^. Luminescence was measured 48 hours later, using the Victor^TM^3 luminometer (Perkin Elmer). Each assay was performed in quadruplicate.

### Statistical analysis

Results were analysed using two-way ANOVA using StataMP 13.0 (Stata Corp. 2013, College Station, TX, USA). A significance level of p < 0.05 was accepted as statistically significant.

## Additional Information

**How to cite this article**: Serena, M. *et al*. HIV-1 Env associates with HLA-C free-chains at the cell membrane modulating viral infectivity. *Sci. Rep.* **7**, 40037; doi: 10.1038/srep40037 (2017).

**Publisher's note:** Springer Nature remains neutral with regard to jurisdictional claims in published maps and institutional affiliations.

## Supplementary Material

Supplementary Information

Supplementary Video S1
