# Corrigendum: HIV-1 Env associates with HLA-C free-chains at the cell membrane modulating viral infectivity

**DOI:** 10.1038/srep46991

**Published:** 2018-05-22

**Authors:** Michela Serena, Francesca Parolini, Priscilla Biswas, Francesca Sironi, Almudena Blanco Miranda, Elisa Zoratti, Maria Teresa Scupoli, Serena Ziglio, Agustin Valenzuela-Fernandez, Davide Gibellini, Maria Grazia Romanelli, Antonio Siccardi, Mauro Malnati, Alberto Beretta, Donato Zipeto

Scientific Reports 7: Article number: 4003710.1038/srep40037; published online: 01 04 2017; updated: 05 22 2018

The original version of this Article contained an error in Figure 7A, where the immunoblot of flotillin from HeLa was a duplicate of the immunoblot of flotillin from HeLa-LAI.

This error has been corrected in the HTML and PDF versions of the Article and the figure has been appropriately delineated. The original images for the immunoblots shown in Figure 7 are now included in the accompanying Supplementary Information file.

