# Supplementary material for: HIV-1 Env associates with HLA-C free-chains at the cell membrane modulating viral infectivity
Source: Sci Rep. 2017 Jan 4;7:40037. doi: 10.1038/srep40037 (PMC5209703; doi:10.1038/srep40037)
Supplement: Supplementary Information [file srep40037-s1.pdf]

HIV-1 Env associates with HLA-C free-chains at the cell membrane modulating viral infectivity

Michela Serena[1], Francesca Parolini[1], Priscilla Biswas[2], Francesca Sironi[2], Almudena Blanco Miranda[1], Elisa Zoratti[3], Maria Teresa Scupoli[3], Serena Ziglio[1,4], Agustin Valenzuela-Fernandez[4], Davide Gibellini[5], Maria Grazia Romanelli[1], Antonio Siccardi[2], Mauro Malnati[2], Alberto Beretta[2], Donato Zipeto[1*]

Supplementary Video S1: Analysis of the localization of Env and HLA-C

All the z-stacks of a cell transfected with Env-Yn and HLA-C-Yc show the complementation of the YFP (green signal), confirming the association between Env and HLA-C in membrane clusters. β_2m is labelled in red.
